# Beam-Hopping-Based Resource Allocation in Integrated Satellite-Terrestrial Networks

**DOI:** 10.3390/s24144699

**Published:** 2024-07-19

**Authors:** Mengying Zhang, Xiumei Yang, Zhiyong Bu

**Affiliations:** 1 Shanghai Institute of Microsystem and Information Technology, Chinese Academy of Sciences, Shanghai 200050, China; xiumei.yang@mail.sim.ac.cn (X.Y.); zhiyong.bu@mail.sim.ac.cn (Z.B.); 2 University of Chinese Academy of Sciences, Beijing 100049, China; 3 Key Laboratory of Wireless Sensor Network and Communications, Chinese Academy of Sciences, Shanghai 200050, China

**Keywords:** integrated satellite-terrestrial network, spectrum sharing, resource allocation, beam-hopping

## Abstract

The integrated satellite-terrestrial network (ISTN) provides a promising solution to achieve high-data-rate and ubiquitous connectivity in next-generation communication networks. Considering the scarce spectrum resources and unevenly distributed traffic demands, we investigate the resource allocation algorithms for ISTNs, where the beam-hopping (BH)-based satellite system and terrestrial systems share the same frequency band. Taking advantage of the scheduling flexibility of BH technology, the dynamical protection zones are constructed to avoid co-channel interference and improve the spectrum efficiency. Since both spectrum efficiency and user fairness are the key optimization indexes in practical systems, two resource allocation problems are formulated to maximize the weighted sum of capacity (MWSC) and maximize the minimum capacity-to-demand ratio (MMCDR) of ISTNs, respectively. By reformulating the problems as mixed-integer linear programming problems, optimal solutions are obtained. To reduce the computational complexity, two greedy suboptimal algorithms are proposed for the MWSC and MMCDR, respectively. The simulation results show that the proposed algorithms achieve higher spectrum efficiency and guarantee fairness between the satellite and terrestrial systems. It is also shown that both the greedy algorithms perform similarly to the optimal algorithms while having much lower complexity.

## 1. Introduction

In order to support emerging applications at any time and any place, sixth-generation (6G) mobile communication networks are expected to provide ubiquitous wireless access services with high data rates [1]. Terrestrial communication systems can meet most of the traffic demands in densely populated areas, but they are unable to provide full wireless coverage in remote and isolated areas because of the lack of infrastructure. Thanks to the advantages of wide coverage and being unlimited by geographical conditions, the satellite communication system can enhance the capacity and support seamless connectivity, especially in areas with adverse environments, such as ocean, desert, and polar regions [2]. Therefore, the integrated satellite-terrestrial network (ISTN) is considered a promising network architecture to satisfy the 6G requirements [3].

With the rapid expansion of both satellite and terrestrial systems, the problem of spectrum scarcity is becoming increasingly severe [4,5]. Spectrum sharing between satellite and terrestrial systems is a feasible way to alleviate the spectrum scarcity issue and enhance the integrated network capacity [6]. However, inter-system resource coordination and management should be carried out to avoid intensive co-channel interference (CCI) in a spectrum sharing scenario.

At present, existing spectrum sharing schemes for ISTNs include database-assisted schemes [7,8], exclusion zone (EZ)-based schemes [9,10], cognitive radio (CR)-based schemes  [5,11,12,13,14,15,16,17], and so on. The database in [7,8] collected the frequency, channel bandwidth, and coverage of each satellite system, and the terrestrial base stations (BSs) could obtain the available frequency band by querying the database. In EZ-based schemes in [9,10], a protection area for the satellite system was constructed to avoid CCI, where terrestrial systems could not transmit on the same frequency band. Considering the highly dynamic topology of the ISTN, database-assisted and EZ-based schemes may lack flexibility, so CR-based schemes have attracted more attention in recent years. To avoid CCI to the primary system, a secondary system can access the frequency band based on the spectrum sensing results or control the interference power below a certain threshold  [5,11,12,13,14,15]. Moreover, a terrestrial BS can act as a cognitive relay to assist the primary satellite system in exchange for spectrum access [16,17]. However, CR-based schemes still face the problems of inaccurate channel state information and spectrum sensing results, as well as a lack of information exchange between the primary and secondary systems, which bring great challenges to satisfy the differentiated and unevenly distributed traffic demands of satellite and terrestrial systems.

With the deepening of satellite-terrestrial integration, it is possible to realize centralized network control and resource management in the ISTN. A software-defined network-based framework for ISTNs was presented in [18], which constructed a unified resource pool for integrated resource management to match the demands of all terminals. The centralized spectrum management server in [19] collected cell load information and set spectrum resource ranges for both terrestrial and non-terrestrial networks to realize coordinated spectrum sharing between them. Compared with isolated spectrum management schemes, integrated resource management is expected to further improve spectrum efficiency and quality of service, as well as suppress CCI between different systems.

Considering the uneven distribution of traffic demands in the ISTN, limited resources should be allocated to each system according to its practical demand. In multi-beam satellite systems, beam-hopping (BH) technology has been used to flexibly provide services to the terminals by changing beam directions in different time slots, which has proved to be more adaptive to unevenly distributed and time-variant traffic demands [20]. BH mechanisms in [20,21,22,23,24,25,26,27,28] dynamically selected illuminated cells and allocated transmission resources according to traffic demands, aiming to maximize system capacity, guarantee user fairness, or improve energy efficiency. However, the spectrum sharing between satellite and terrestrial systems has not been considered in these works. A BH-based resource allocation scheme for cognitive satellite communication system was proposed in [14], where the frequency band used by each beam depended on the spectrum sensing results from the terminals. In the cognitive satellite-terrestrial network in [15], the BH-based primary satellite system optimized the BH pattern and beam power to improve the weighted capacity–request ratio, while the secondary terrestrial system applied the spectrum detection mechanism to sense the satellite system. Nevertheless, the integrated resource allocation for spectrum sharing between BH-based satellite systems and terrestrial systems has not been investigated, and the approaches in the prior work cannot be directly applied to jointly optimize the performance of both the satellite and terrestrial systems.

In this paper, we investigate resource allocation for the ISTN, which consists of a BH-based satellite system and multiple terrestrial systems. It is assumed that the satellite system and the terrestrial systems share the same frequency band, while all spot beams of the satellite also reuse the full bandwidth to further improve the spectrum efficiency. The centralized network control center (NCC) in the ISTN is responsible for resource allocation and management to enhance system capacity as well as satisfy traffic demands of all the satellite and terrestrial systems. The main contributions of this work are summarized as follows:

- A BH-based spectrum sharing scheme for the ISTN is presented, which constructs the dynamical protection zones according to the beam allocation pattern of the satellite. Compared with the conventional static EZ, the dynamical protection zones can improve the reusability of the frequency resources while avoiding the CCI between the satellite and terrestrial systems.
- Based on the spectrum sharing scheme, resource allocation problems are formulated to maximize the weighted sum of capacity of the ISTN and guarantee fairness among the systems, respectively. To obtain optimal solutions, these two optimization problems are reformulated as mixed integer linear programming (MILP) problems based on beam allocation patterns.
- To accelerate the resource allocation process, two greedy suboptimal algorithms are proposed to solve problems with low complexity, which are more suitable for real-time resource scheduling in the ISTN.
- Simulations are carried out to evaluate the performance of the proposed algorithms. The simulation results show that the proposed resource allocation algorithms achieve higher spectrum efficiency and better matching between transmission capacity and traffic demands of both the satellite and terrestrial systems. It is also shown that both greedy algorithms with low complexity can achieve performance close to that of the optimal algorithms.

The rest of this paper is organized as follows. Section 2 describes the scenario and the spectrum sharing scheme of the ISTN. The downlink transmission models for the satellite and terrestrial systems are also provided in this section. Resource allocation problems in the ISTN are formulated in Section 3. Optimal and greedy suboptimal algorithms for the two resource allocation problems are proposed in Section 4 and Section 5, respectively. Section 6 presents the simulation results, and Section 7 concludes this paper.

## 2. System Model

### 2.1. Scenario

**Figure 1:**
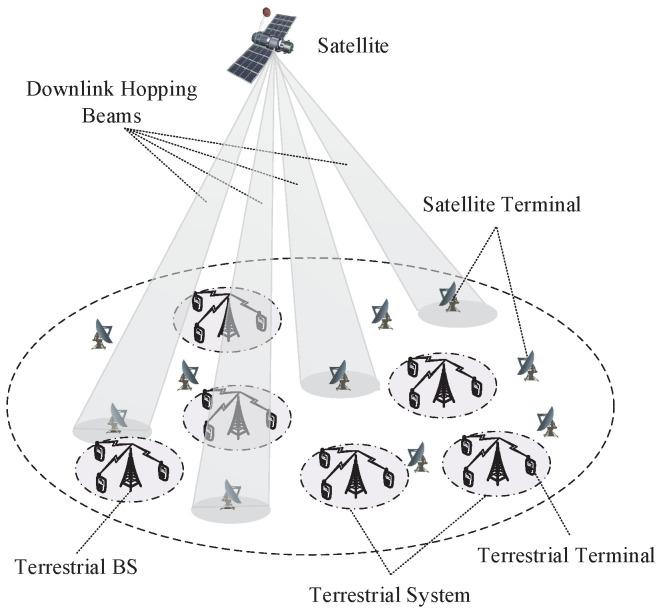
The downlink transmission scenario in an ISTN.

Consider the downlink transmission scenario in an ISTN as shown in Figure 1. The satellite system and multiple terrestrial systems are controlled by the same NCC and share the same frequency band to improve the spectrum efficiency.

The satellite system consists of a satellite with the orbit height of $h_{s}$ and $N_{sat}$ satellite terminals indexed by the set $N_{sat}=\left\{1,\ldots ,N_{sat}\right\}$. The satellite is equipped with a phased array antenna [29], which can form $N_{b}$ spot beams to serve the satellite terminals via a BH manner. A BH time window consists of $N_{slot}$ time slots indexed by the set $\Gamma =\{1,\ldots ,N_{slot}\}$, where the duration of a time slot is denoted by $T_{slot}$. Each spot beam can serve one satellite terminal in each time slot. Therefore, at most $N_{b}$ satellite terminals can be served by the satellite system in each time slot. All the spot beams reuse the full transmission bandwidth.

There are $N_{ter}$ terrestrial systems within the footprint of the satellite, which can be indexed by the set $N_{ter}=\left\{1,\ldots ,N_{ter}\right\}$. A terrestrial BS is located in the center of each terrestrial system and provides services to the terrestrial terminals in this system. The locations of the terrestrial BSs are modeled by the Matern hardcore process (MHP) [30], which ensures a minimum separation distance δ between different terrestrial BSs. It is assumed that δ is quite larger than the cell radius of the terrestrial systems, so all the terrestrial systems can reuse the same frequency band without the inter-BS CCI.

### 2.2. Spectrum Sharing Scheme in ISTN

As mentioned in Section 1, EZ-based spectrum sharing schemes have been widely used to limit the CCI and ensure the isolation between satellite beams and terrestrial BSs in the ISTN. However, the conventional static EZ is inapplicable to the low Earth orbit (LEO) or medium Earth orbit (MEO) satellite systems with high mobility, and the oversized EZ may reduce the available resources of the terrestrial systems and degrade the spectrum efficiency [10].

Considering the high flexibility of BH technology, dynamical protection zones can be constructed to avoid inter-system CCI and improve the reusability of the frequency resources. In each time slot, protection zones are only constructed around the satellite terminals served by the spot beams. Except for these served satellite terminals, the satellite terminals do not need to be protected since they will not receive the downlink signals from the satellite in this time slot. This means that the protection zones change dynamically in different time slots, and the terrestrial BSs that can perform transmission also change accordingly.

Define $d_{pro}^{st}$ as the inter-system protection distance between the satellite terminal and the terrestrial BS working in the same time slot. Assuming that the *i*-th satellite terminal ($i\in N_{sat}$) is served by the satellite system in the time slot t∈Γ, the terrestrial BS in the *j*-th terrestrial system ($j\in N_{ter}$) cannot perform transmission if it is located in the protection zone of the *i*-th satellite terminal, i.e., the distance $d_{i,j}^{st}$ between the *j*-th terrestrial BS and the *i*-th satellite terminal is less than $d_{pro}^{st}$. From another perspective, if the *j*-th terrestrial BS is allowed to perform transmission in the time slot *t*, there is no served satellite terminal within a distance of $d_{pro}^{st}$ around the terrestrial BS. To avoid inter-system CCI, the size of the EZ is generally larger than the size of the satellite beam [10]. Therefore, the minimum protection distance in the BH-based spectrum sharing scheme should be the coverage radius of the spot beam corresponding to the 3 dB beam width.

Corresponding to the BH-based spectrum sharing scheme, the procedure for resource allocation in the ISTN can be described as follows.

First, the NCC collects the locations and the transmission capacity requirements of all the satellite terminals and terrestrial BSs. To ensure security, the accuracy of the location information and the capacity requirements can be properly reduced through the fuzzification process, and the security mechanisms and encryption schemes can be adopted to achieve secure transmission.

Based on the acquired information, the NCC allocates the time slots in a BH time window to the satellite terminals and the terrestrial BSs according to the capacity requirements. Let $x_{i,t}\in \left\{0,1\right\}$ and $y_{j,t}\in \left\{0,1\right\}$ indicate whether the time slot t∈Γ is allocated to the *i*-th satellite terminal ($i\in N_{sat}$) and the *j*-th terrestrial BS ($j\in N_{ter}$), respectively. If the time slot *t* is allocated to both the *i*-th satellite terminal and the *j*-th terrestrial BS, i.e., $x_{i,t}=y_{j,t}=1$, the distance $d_{i,j}^{st}$ between them should be larger than or equal to $d_{pro}^{st}$.

Then, the resource allocation results are transmitted to the satellite and the terrestrial BSs. Specifically, the resource allocation results for the satellite system indicate the satellite terminals served by the spot beams in each time slot, while the results for each terrestrial system indicate the time slots allowed for transmission in a BH time window.

After receiving the resource allocation results, the satellite provides services to the corresponding satellite terminals in each time slot, and the terrestrial BSs perform the downlink transmission in the allocated time slots.

### 2.3. Downlink Transmission Model

Based on the above scenario and spectrum sharing scheme, the received signal-to-interference-plus-noise ratio (SINR) of the *i*-th satellite terminal ($i\in N_{sat}$) in time slot t∈Γ can be expressed as

(1) $$\varrho_{i,t}^{sat}=\frac{\alpha_{i}^{sat}P_{b}^{sat}}{N_{0}^{sat}+\sum_{k=1,k\neq i}^{N_{sat}}x_{k,t}I_{k,i}^{sat}},$$

where $\alpha_{i}^{sat}$ denotes the channel gain between the satellite and the *i*-th satellite terminal, $P_{b}^{sat}$ denotes the transmit power of each satellite beam, $N_{0}^{sat}$ denotes the noise power, and $I_{k,i}^{sat}$ denotes the interference power from the spot beam pointing to the *k*-th satellite terminal. The noise power $N_{0}^{sat}=KT_{n}W$, where *K* is the Boltzmann constant, $T_{n}$ is the receiver noise temperature, and *W* is the transmission bandwidth. Because of the introduction of the inter-system protection distance, CCI from the terrestrial systems to the satellite terminal can be neglected.

It is assumed that the receiving antenna of the satellite terminal can keep pointing to the satellite and the channel gain remains unchanged in a BH time window. Then, $\alpha_{i}^{sat}$ can be expressed as

(2) $$\alpha_{i}^{sat}=G_{T,0}^{sat}G_{R,0}^{sat}/L_{i}^{sat},$$

where $G_{T,0}^{sat}$ is the peak transmitting antenna gain of the satellite, $G_{R,0}^{sat}$ is the peak receiving antenna gain of the satellite terminal, and $L_{i}^{sat}$ is the free space propagation loss between the satellite and the *i*-th satellite terminal. The free space propagation loss is a function of the distance between the transmitter and the receiver. Specifically, $L_{i}^{sat}$ can be written as

(3) $$L_{i}^{sat}={\left(\frac{4\pi d_{i}^{sat}}{c/f}\right)}^{2},$$

where $d_{i}^{sat}$ is the distance between the satellite and the *i*-th satellite terminal, *c* is the velocity of light, and *f* is the carrier frequency.

Because of full frequency multiplexing, the inter-beam interference power $I_{k,i}^{sat}$ (k≠i) can be expressed as

(4) $$I_{k,i}^{sat}=G_{T}^{sat}\left(\theta_{k,i}\right)G_{R,0}^{sat}P_{b}^{sat}/L_{i}^{sat},$$

where $G_{T}^{sat}\left(\theta_{k,i}\right)$ denotes the transmitting antenna gain of the satellite in the direction of angle $\theta_{k,i}$, which is given by [31]

(5) $$G_{T}^{sat}\left(\theta_{k,i}\right)=G_{T,0}^{sat}{\left(\frac{J_{1}\left(u\left(\theta_{k,i}\right)\right)}{2u(\theta_{k,i})}+36\frac{J_{3}\left(u\left(\theta_{k,i}\right)\right)}{u{\left(\theta_{k,i}\right)}^{3}}\right)}^{2},$$

where $\theta_{k,i}$ is the off-axis angle of the *i*-th satellite terminal from the bore sight of the satellite beam pointing to the *k*-th satellite terminal, $J_{1}\left(\cdot \right)$ and $J_{3}\left(\cdot \right)$ are the first-kind Bessel function of orders 1 and 3, respectively, and $u(\theta_{k,i})$ is expressed as [31]

(6) $$u\left(\theta_{k,i}\right)=2.07123\frac{\sin(\theta_{k,i})}{\sin(\theta_{3dB})},$$

where $\theta_{3dB}$ is the 3 dB beam width of the transmitting antenna. It should be noted that as $\theta_{k,i}$ decreases, the transmitting antenna gain $G_{T}^{sat}\left(\theta_{k,i}\right)$ and the inter-beam interference power $I_{k,i}^{sat}$ increases, which may degrade the SINR of the *i*-th satellite terminal.

Based on Shannon’s theorem, the transmission capacity of the *i*-th satellite terminal in a BH time window can be expressed as

(7) $$C_{i}^{sat}=\sum_{t=1}^{N_{slot}}x_{i,t}WT_{slot}\log_{2}\left(1+\varrho_{i,t}^{sat}\right).$$

In each terrestrial system, the terrestrial BS may provide services to multiple terrestrial terminals. Since the locations of the terrestrial terminals may change from time to time, the worst case is considered where all terrestrial terminals are located at the cell edge of the terrestrial system. Therefore, the worst-case transmission capacity of the *j*-th terrestrial system in each time slot can be expressed as

(8) $$c_{j}^{ter}=WT_{slot}\log_{2}\left(1+\frac{\beta_{j}^{ter}P_{j}^{ter}}{N_{0}^{ter}}\right),$$

where $\beta_{j}^{ter}$ denotes the worst-case channel gain between the terrestrial BS and the terrestrial terminals in the *j*-th terrestrial system, $P_{j}^{ter}$ denotes the transmit power of the *j*-th terrestrial BS, and $N_{0}^{ter}$ denotes the noise power.

It is assumed that the channel gain remains unchanged in a BH time window. $\beta_{j}^{ter}$ can be expressed as

(9) $$\beta_{j}^{ter}=G_{T}^{ter}G_{R}^{ter}/\left(L_{j}^{ter}\eta_{j}^{ter}\right),$$

where $G_{T}^{ter}$ is the transmitting antenna gain of the terrestrial BS, $G_{R}^{ter}$ is the receiving antenna gain of the terrestrial terminals, $L_{j}^{ter}$ is the path loss between the terrestrial BS and the terrestrial terminal at the cell edge, and $\eta_{j}^{ter}$ is the shadowing loss. The path loss models and the shadowing standard deviations in different scenarios are provided in [32].

On this basis, the worst-case transmission capacity of the *j*-th terrestrial system in a BH time window is

(10) $$C_{j}^{ter}=\sum_{t=1}^{N_{slot}}y_{j,t}c_{j}^{ter}.$$

## 3. Problem Formulation

As mentioned in Section 2.2, the NCC allocates the time slots in a BH time window to the satellite terminals and the terrestrial BSs. The objective of resource allocation depends on the practical needs of the operators, such as optimizing system spectrum efficiency or guaranteeing fairness between the satellite and terrestrial systems. In this section, resource allocation problems are formulated from the following two perspectives, respectively:

- Maximizing the weighted sum of capacity (MWSC) to improve the spectrum efficiency of the ISTN.
- Maximizing the minimum capacity-to-demand ratio (MMCDR) to guarantee fairness in the ISTN.

### 3.1. Problem Formulation for MWSC

To improve the spectrum efficiency of the ISTN, the resource allocation problem can be formulated as

(11) $$\begin{array}{cc} P1:\max_{\left\{x_{i,t}\right\},\left\{y_{j,t}\right\}} & \sum_{i=1}^{N_{sat}}\min\left(C_{i}^{sat},R_{i}^{sat}\right)+\gamma \sum_{j=1}^{N_{ter}}C_{j}^{ter} \end{array}$$

(12) $$\begin{array}{cc} s.t.\; & x_{i,t}\in \left\{0,1\right\},\forall i\in N_{sat},t\in \Gamma \end{array}$$

(13) $$\begin{array}{cc} \; & y_{j,t}\in \left\{0,1\right\},\forall j\in N_{ter},t\in \Gamma \end{array}$$

(14) $$\begin{array}{cc} \; & \sum_{i=1}^{N_{sat}}x_{i,t}\leq N_{b},\forall t\in \Gamma \end{array}$$

(15) $$\begin{array}{cc} \; & C_{j}^{ter}\geq R_{j,min}^{ter},\forall j\in N_{ter} \end{array}$$

(16) $$\begin{array}{cc} \; & d_{i,j}^{st}\geq d_{pro}^{st},\mathrm{if}\;x_{i,t}=y_{j,t}=1,\forall i\in N_{sat},j\in N_{ter},t\in \Gamma , \end{array}$$

where $R_{i}^{sat}$ denotes the requested capacity of the *i*-th satellite terminal in a BH time window, $R_{j,min}^{ter}$ denotes the minimum required capacity of the *j*-th terrestrial system in a BH time window, and γ is used to control the weight of the capacity of the satellite and terrestrial systems. To avoid wasting the limited energy of the satellite, the actual transmission capacity provided to the *i*-th satellite terminal should not exceed its requested capacity, so the actual capacity depends on the minimum value of $C_{i}^{sat}$ and $R_{i}^{sat}$. In practical systems, if $C_{i}^{sat}$ is larger than $R_{i}^{sat}$, the satellite can either reduce the transmit power or shorten the transmission time to make the actual transmission capacity equal to $R_{i}^{sat}$. Constraints (12) and (13) define the optimization variables $x_{i,t}$ and $y_{j,t}$, respectively, $\forall i\in N_{sat},j\in N_{ter},t\in \Gamma$, which indicate the resource allocation results in each time slot. Constraint (14) means that at most $N_{b}$ satellite terminals can be served in each time slot. Constraint (15) states that the transmission capacity of the *j*-th terrestrial system should not be lower than its minimum required capacity. Constraint (16) represents the protection distance constraint between the satellite and terrestrial systems.

### 3.2. Problem Formulation for MMCDR

The capacity to demand ratio (CDR) is defined as the ratio of the transmission capacity to the average traffic demand of each satellite terminal or terrestrial system. To maximize the minimum CDR in the ISTN, the resource allocation problem can be formulated as

(17) $$P2:\max_{\left\{x_{i,t}\right\},\left\{y_{j,t}\right\}}\min\left(\min_{i\in N_{sat}}\left(\frac{C_{i}^{sat}}{D_{i}^{sat}}\right),\min_{j\in N_{ter}}\left(\frac{C_{j}^{ter}}{D_{j}^{ter}}\right)\right)$$

s.t.(12),(13),(14),(16),

where $D_{i}^{sat}$ denotes the average traffic demand of the *i*-th satellite terminal in a BH time window and $D_{j}^{ter}$ denotes the average traffic demand of the *j*-th terrestrial system in a BH time window, where the average traffic demand can be obtained by analyzing the historical data of the capacity requirements. This problem aims to optimize the minimum CDR of the satellite terminals and the terrestrial systems so that the transmission resources and the traffic demands can achieve better matching and fairness can be guaranteed.

Both the above two optimization problems, P1 and P2, are binary-integer nonlinear programming problems. Directly solving the optimal solutions of these problems leads to extremely high computational complexity. To obtain optimal solutions efficiently, we reformulate these problems as MILP problems and solve them via classical optimization algorithms. Furthermore, two greedy suboptimal algorithms are also proposed for P1 and P2, respectively, and can achieve desirable performance with much lower complexity.

## 4. Resource Allocation Algorithms for MWSC

In this section, we first present a reformulation of P1 as an MILP problem, which helps obtain the optimal solutions efficiently, and then propose a greedy suboptimal algorithm with low computational complexity.

### 4.1. Optimal Algorithm

At the beginning of the reformulation, all feasible beam allocation patterns that satisfy the constraint (14) should be found. In each time slot, the satellite selects *n* satellite terminals ($0\leq n\leq \min(N_{b},N_{sat})$) from $N_{sat}$ satellite terminals to provide services. Thus, the total number of beam allocation patterns is

(18) MBAP=∑n=0min(Nb,Nsat)Nsatn.

Form the $N_{sat}\times M_{BAP}$ beam allocation pattern matrix U, where the rows of the matrix correspond to the satellite terminal indices and the columns correspond to the beam allocation pattern indices. For the *m*-th beam allocation pattern (the *m*-th column of U), element $u_{i,m}\in \left\{0,1\right\}$ of the *i*-th row indicates whether the *i*-th satellite terminal is served by a spot beam. If $u_{i,m}=1$, the *i*-th satellite terminal is served when the *m*-th beam allocation pattern is applied.

Corresponding to the beam allocation pattern matrix, the $N_{ter}\times M_{BAP}$ transmission pattern matrix V for the terrestrial systems can be obtained as follows. The rows of the matrix V correspond to the terrestrial BS indices, and the columns correspond to the beam allocation pattern indices. For the *m*-th beam allocation pattern (the *m*-th column of V), element $v_{j,m}\in \left\{0,1\right\}$ of the *j*-th row indicates whether the *j*-th terrestrial BS can perform downlink transmission. Based on the constraint (16), the value of $v_{j,m}$ is determined as

(19) $$v_{j,m}=\left\{\begin{array}{cc} 1, & \mathrm{if}\;\min_{i\in \Phi_{m}}d_{i,j}^{st}\geq d_{pro}^{st}\\ 0, & \mathrm{if}\;\min_{i\in \Phi_{m}}d_{i,j}^{st}<d_{pro}^{st} \end{array}\right.,$$

where the set $\Phi_{m}=\left\{k\in N_{sat}|u_{k,m}=1\right\}$ indicates the satellite terminals that are served by the spot beams when the *m*-th beam allocation pattern is applied.

After obtaining matrices U and V, the transmission capacity of the satellite and terrestrial systems associated with each beam allocation pattern can be determined. In each time slot, when the *m*-th beam allocation pattern is applied, the capacity of the *i*-th satellite terminal is

(20) $$\begin{array}{c} {\hat{c}}_{i,m}^{sat}=u_{i,m}WT_{slot}\log_{2}\left(1+\frac{\alpha_{i}^{sat}P_{b}^{sat}}{N_{0}^{sat}+\sum_{k=1,k\neq i}^{N_{sat}}u_{k,m}I_{k,i}^{sat}}\right), \end{array}$$

and the capacity of the *j*-th terrestrial system is

(21) $${\hat{c}}_{j,m}^{ter}=v_{j,m}c_{j}^{ter}.$$

Let the non-negative integer $\mu_{m}$ denote the number of times slots in which the *m*-th beam allocation pattern is applied within a BH time window. Then, introduce the auxiliary variables $\zeta_{i}$, $\forall i\in N_{sat}$, which implies the actual capacity of the *i*-th satellite terminal. The optimization problem P1 can be reformulated as the following MILP problem.

(22) $$\begin{array}{cc} P1^{\prime }:\max_{\left\{\mu_{m}\right\}} & \sum_{i=1}^{N_{sat}}\zeta_{i}+\gamma \sum_{m=1}^{M_{BAP}}\left(\mu_{m}\sum_{j=1}^{N_{ter}}{\hat{c}}_{j,m}^{ter}\right) \end{array}$$

(23) $$\begin{array}{cc} s.t. & \zeta_{i}\leq \sum_{m=1}^{M_{BAP}}\mu_{m}{\hat{c}}_{i,m}^{sat},\forall i\in N_{sat} \end{array}$$

(24) $$\begin{array}{cc} \; & \zeta_{i}\leq R_{i}^{sat},\forall i\in N_{sat} \end{array}$$

(25) $$\begin{array}{cc} \; & \sum_{m=1}^{M_{BAP}}\mu_{m}{\hat{c}}_{j,m}^{ter}\geq R_{j,min}^{ter},\forall j\in N_{ter} \end{array}$$

(26) $$\begin{array}{cc} \; & \sum_{m=1}^{M_{BAP}}\mu_{m}=N_{slot} \end{array}$$

(27) $$\begin{array}{cc} \; & \mu_{1},\ldots ,\mu_{M_{BAP}}\;\mathrm{are}\mathrm{non}-\mathrm{negative}\mathrm{integer}. \end{array}$$

Constraints (23) and (24) mean that the value of $\zeta_{i}$ depends on the minimum value of $C_{i}^{sat}$ and $R_{i}^{sat}$. Constraint (25) is equivalent to Constraint (15). Constraint (26) indicates that the sum of the time slot number for each beam allocation pattern should be equal to $N_{slot}$.

The optimal solution of the MILP problem P1’ can be obtained by classical optimization algorithms, such as the branch-and-bound algorithm [33].

### 4.2. Greedy Suboptimal Algorithm

The optimal algorithm in Section 4.1 can provide an optimal solution to improve the spectrum efficiency of the ISTN. However, the number of optimization variables increases dramatically with $N_{sat}$ and $N_{b}$, which brings obstacles to real-time resource allocation because of the high computational complexity. Therefore, a greedy suboptimal algorithm is proposed to solve P1 with low complexity.

To satisfy constraint (15), the resource guarantee factor *Q* is defined in the greedy algorithm. The value of *Q* is an integer from 0 to $N_{ter}$, which implies the priority of resource allocation for the terrestrial systems. If Q=0, the capacity requirements of the terrestrial systems are not considered when the time slots are allocated to the satellite terminals, so the flexibility of resource allocation is maximized. If $Q=N_{ter}$, time slots are allocated to all terrestrial BSs in priority to meet the minimum capacity requirements.

For a certain value of *Q*, the main idea of the greedy algorithm can be described as follows. For each time slot in a BH time window, select *Q* terrestrial BSs with the maximum remaining capacity requirements and allow them to perform transmission in this time slot. Then, subject to constraints (14) and (16), the time slot is allocated to at most $N_{b}$ satellite terminals with higher remaining capacity requirements. Finally, whether the remaining terrestrial BSs can perform transmission is determined according to the resource allocation results of the satellite terminals.

On the other hand, as mentioned in Section 2.3, the transmission capacity of each satellite terminal is related to the inter-beam CCI. When multiple adjacent satellite terminals are served simultaneously, CCI will significantly degrade the SINR of these satellite terminals. To avoid CCI, an inter-beam distance threshold $d_{th}^{sat}$ is introduced to ensure isolation among the spot beams. If both the *i*-th and *k*-th satellite terminals are served in the same time slot, the distance $d_{i,k}^{sat}$ between them should not be less than $d_{th}^{sat}$. When $d_{th}^{sat}$ is large enough, the inter-beam interference power is far less than the noise power $N_{0}^{sat}$ [23,24]. Thus, inter-beam CCI can be neglected, and the transmission capacity of the *i*-th satellite terminal in each time slot can be expressed as

(28) $${\tilde{c}}_{i}^{sat}=x_{i,t}WT_{slot}\log_{2}\left(1+\frac{\alpha_{i}^{sat}P_{b}^{sat}}{N_{0}^{sat}}\right).$$

The above simplification can help further reduce the complexity of solving the resource allocation problem.

On this basis, the greedy algorithm for MWSC is summarized in Algorithm 1. At the beginning of the algorithm, the resource guarantee factor *Q* is initialized to 0 (line 1). If the minimum required capacity of all the terrestrial systems is achieved after the resource allocation process (lines 2–25), the algorithm terminates (lines 26–28); otherwise, repeat the resource allocation process with Q=Q+1 until $Q=N_{ter}$.

During the resource allocation process, $R_{i,re}^{sat}$ and $R_{j,re}^{ter}$ denote the remaining requested capacity of the *i*-th satellite terminal and the remaining required capacity of the *j*-th terrestrial system, which are initialized to $R_{i}^{sat}$ and $R_{j,min}^{ter}$ (lines 2–3), respectively. For each time slot t∈Γ, the resource allocation results can be obtained as follows:

- Initialize the candidate sets $\Omega_{s}$ and $\Omega_{t}$ (lines 5–6). $\Omega_{s}$ indicates the candidate satellite terminals that can be served in time slot *t*. The satellite terminals without remaining requested capacity should be removed from $\Omega_{s}$. $\Omega_{t}$ indicates the candidate terrestrial BSs that can perform transmission in time slot *t*, which is initialized to $N_{ter}$.
- Allocate the time slot *t* to at most *Q* terrestrial BSs (lines 7–13). While the number of selected terrestrial BSs is less than *Q* and the maximum $R_{j,re}^{ter}$ of the candidate terrestrial BSs is greater than 0, the time slot *t* is allocated to the terrestrial BS $j^{*}\in \Omega_{t}$ with the maximum $R_{j,re}^{ter}$ in each iteration. Then, $R_{j^{*},re}^{ter}$ is updated, and the terrestrial BS $j^{*}$ is removed from $\Omega_{t}$. To avoid inter-system CCI, $\Omega_{s}$ is updated based on the inter-system protection distance $d_{pro}^{st}$.
- Allocate the time slot *t* to at most $N_{b}$ satellite terminals (lines 14–20). While $\Omega_{s}$ is not empty and the number of served satellite terminals is less than $N_{b}$, the time slot *t* is allocated to the satellite terminal $i^{*}\in \Omega_{s}$ with the maximum $R_{i,re}^{sat}$ in each iteration. Then, $R_{i^{*},re}^{sat}$ is updated. To avoid inter-beam CCI and inter-system CCI, $\Omega_{s}$ and $\Omega_{t}$ are updated based on $d_{th}^{sat}$ and $d_{pro}^{st}$, respectively.
- Allocate the time slot *t* to the remaining terrestrial BSs in $\Omega_{t}$ (lines 21–24) since they are outside the protection zones of the served satellite terminals.

Repeat the above process until all time slots are allocated, and then decide whether the algorithm can terminate.
**Algorithm 1** Greedy algorithm for MWSC.**Input:** $\left\{R_{i}^{sat}\right\}$, $\left\{R_{j,min}^{ter}\right\}$, $\left\{{\tilde{c}}_{i}^{sat}\right\}$, $\left\{c_{j}^{ter}\right\}$, $\left\{d_{i,j}^{st}\right\}$, $\left\{d_{i,k}^{sat}\right\}$, $\forall i,k\in N_{sat},j\in N_{ter}$ **Output:** $\left\{x_{i,t}\right\},\left\{y_{j,t}\right\},\forall i\in N_{sat},j\in N_{ter},t\in \Gamma$  1:**for** each $Q=0,\cdots ,N_{ter}$ **do**  2:   $x_{i,t}\leftarrow 0,R_{i,re}^{sat}\leftarrow R_{i}^{sat}$, $\forall i\in N_{sat},t\in \Gamma$  3:   $y_{j,t}\leftarrow 0,R_{j,re}^{ter}\leftarrow R_{j,min}^{ter}$, $\forall j\in N_{ter},t\in \Gamma$  4:   **for** each t∈Γ **do**  5:     $\Omega_{s}\leftarrow \left\{i\in N_{sat}|R_{i,re}^{sat}>0\right\}$  6:     $\Omega_{t}\leftarrow N_{ter}$  7:     **while** $\sum_{j=1}^{N_{ter}}y_{j,t}<Q$ and $\max_{j\in \Omega_{t}}\left\{R_{j,re}^{ter}\right\}>0$ **do**  8:        $j^{*}\leftarrow$arg$\max_{j\in \Omega_{t}}\left\{R_{j,re}^{ter}\right\}$  9:        $y_{j^{*},t}\leftarrow 1$10:        $R_{j^{*},re}^{ter}\leftarrow R_{j^{*},re}^{ter}-c_{j^{*}}^{ter}$11:        $\Omega_{t}\leftarrow \Omega_{t}-\left\{j^{*}\right\}$12:        $\Omega_{s}\leftarrow \left\{i\in \Omega_{s}|d_{i,j^{*}}^{st}\geq d_{pro}^{st}\right\}$13:     **end while**14:     **while** $\Omega_{s}\neq \emptyset$ and $\sum_{i=1}^{N_{sat}}x_{i,t}<N_{b}$ **do**15:        $i^{*}\leftarrow$arg$\max_{i\in \Omega_{s}}\left\{R_{i,re}^{sat}\right\}$16:        $x_{i^{*},t}\leftarrow 1$17:        $R_{i^{*},re}^{sat}\leftarrow R_{i^{*},re}^{sat}-{\tilde{c}}_{i^{*}}^{sat}$18:        $\Omega_{s}\leftarrow \left\{k\in \Omega_{s}|d_{i^{*},k}^{sat}\geq d_{th}^{sat}\right\}$19:        $\Omega_{t}\leftarrow \left\{j\in \Omega_{t}|d_{i^{*},j}^{st}\geq d_{pro}^{st}\right\}$20:     **end while**21:     **for** each $j\in \Omega_{t}$ **do**22:        $y_{j,t}\leftarrow 1$23:        $R_{j,re}^{ter}\leftarrow R_{j,re}^{ter}-c_{j}^{ter}$24:     **end for**25:   **end for**26:   **if** $\max_{j\in N_{ter}}\left\{R_{j,re}^{ter}\right\}\leq 0$ **then**27:     break28:   **end if**29:**end for**

The computational complexity of the greedy algorithm for MWSC mainly depends on the number of operations in calculating the transmission capacity of the satellite terminals and the terrestrial systems, as well as the number of operations in updating the candidate sets based on the inter-system and inter-beam distance thresholds. Specifically, it requires $O(N_{sat})$ operations to calculate $c_{i}^{sat}$ for all satellite terminals and $O(N_{ter})$ operations to calculate $c_{j}^{ter}$ for all terrestrial systems before the resource allocation. In the main body of the algorithm, there are at most $N_{ter}+1$ resource allocation processes since *Q* is an integer from 0 to $N_{ter}$. In the resource allocation process with the resource guarantee factor *Q*, it requires at most $O(\left(Q+2N_{b}\right)N_{slot})$ operations to update the candidate sets $\Omega_{s}$ and $\Omega_{t}$ based on $d_{pro}^{st}$ and $d_{th}^{sat}$ when $N_{slot}$ time slots are allocated. Therefore, the worst-case complexity is $O(\left(N_{ter}+1\right)\left(N_{ter}/2+2N_{b}\right)N_{slot})$. In practice, the computational complexity is much less than this because the algorithm terminates if the minimum required capacity of all the terrestrial systems is met.

## 5. Resource Allocation Algorithms for MMCDR

In this section, the optimization problem P2 is reformulated as an MILP problem in a way similar to Section 4.1, and then, a greedy suboptimal algorithm is proposed to solve P2 with low computational complexity.

### 5.1. Optimal Algorithm

Before the reformulation, the beam allocation pattern matrix U and the transmission pattern matrix V are formed by using the same approach as in Section 4.1. Then, corresponding to each beam allocation pattern, the transmission capacity of each satellite terminal and each terrestrial system can be calculated by (20) and (21), respectively.

Introduce the auxiliary variable ξ, which represents the minimum CDR in the ISTN. Then, the optimization problems P2 for MMCDR can be reformulated as the following MILP problem.

(29) $$\begin{array}{cc} P2^{\prime }:\max_{\left\{\mu_{m}\right\}} & \;\xi \end{array}$$

(30) $$\begin{array}{cc} s.t. & \xi \leq \sum_{m=1}^{M_{BAP}}\mu_{m}{\hat{c}}_{i,m}^{sat}/D_{i}^{sat},\forall i\in N_{sat} \end{array}$$

(31) $$\begin{array}{cc} \; & \xi \leq \sum_{m=1}^{M_{BAP}}\mu_{m}{\hat{c}}_{j,m}^{ter}/D_{j}^{ter},\forall j\in N_{ter}\\ \; & \left(26),(27\right). \end{array}$$

Constraints (30) and (31) mean that the value of ξ depends on the minimum CDR of the satellite terminals and the terrestrial systems.

The optimal solution of the MILP problem P2’ can also be obtained by the branch-and-bound algorithm.

### 5.2. Greedy Suboptimal Algorithm

As mentioned in Section 4.2, the computational complexity in solving the MILP problem is nonnegligible. Therefore, a greedy suboptimal algorithm with low complexity is proposed to solve problem P2.

To avoid CCI, the inter-beam distance threshold $d_{th}^{sat}$ is also used in the greedy algorithm for MMCDR, and the transmission capacity of each satellite terminal in each time slot can be calculated by (28).

The greedy algorithm for MMCDR is summarized in Algorithm 2. For each time slot in a BH time window, the satellite terminal or the terrestrial system with the minimum CDR will have priority in time slot allocation.

At the beginning of the algorithm, the resource allocation results and the transmission capacity of the satellite and terrestrial systems are initialized (lines 1–2), respectively.

For each time slot t∈Γ, the resource allocation results can be obtained as follows. Initialize the candidate sets $\Omega_{s}$ and $\Omega_{t}$ (line 4). In each iteration, if $\Omega_{s}$ is not empty and the number of served satellite terminals in time slot *t* is less than $N_{b}$, the time slot *t* is allocated to one of the candidate satellite terminals or one of the candidate terrestrial BSs as follows:

- If the minimum CDR of the satellite terminals is less than or equal to that of the terrestrial BSs, the time slot *t* is allocated to one of the candidate satellite terminals (lines 6–12). The set $\Theta_{s}$ indicates the satellite terminals with the minimum CDR in $\Omega_{s}$. The time slot *t* is allocated to the satellite terminal $i^{*}\in \Theta_{s}$ with the maximum $D_{i}^{sat}$. Then, $C_{i^{*}}^{sat}$ is updated. To avoid inter-beam and inter-system CCI, $\Omega_{s}$ and $\Omega_{t}$ are updated based on $d_{th}^{sat}$ and $d_{pro}^{st}$, respectively.
- Otherwise, the time slot *t* is allocated to one of the candidate terrestrial BSs (lines 13–20). The set $\Theta_{t}$ indicates the terrestrial BSs with the minimum CDR in $\Omega_{t}$. The time slot *t* is allocated to the terrestrial BS $j^{*}\in \Theta_{t}$ with the maximum $D_{j}^{ter}$. Then, $C_{j^{*}}^{ter}$ is updated, and the terrestrial BS $j^{*}$ is removed from $\Omega_{t}$. To avoid inter-system CCI, $\Omega_{s}$ is updated based on $d_{pro}^{st}$.

After determining the served satellite terminals in time slot *t*, the time slot *t* is allocated to the remaining terrestrial BSs in $\Omega_{t}$, and their transmission capacity is updated accordingly (lines 22–25).

Repeat the above process until all time slots are allocated.
**Algorithm 2** Greedy algorithm for MMCDR.**Input:** $\left\{D_{i}^{sat}\right\}$, $\left\{D_{j}^{ter}\right\}$, $\left\{{\tilde{c}}_{i}^{sat}\right\}$, $\left\{c_{j}^{ter}\right\}$, $\left\{d_{i,j}^{st}\right\}$, $\left\{d_{i,k}^{sat}\right\}$, $\forall i,k\in N_{sat},j\in N_{ter}$ **Output:** $\left\{x_{i,t}\right\},\left\{y_{j,t}\right\},\forall i\in N_{sat},j\in N_{ter},t\in \Gamma$  1:$x_{i,t}\leftarrow 0,C_{i}^{sat}\leftarrow 0$, $\forall i\in N_{sat},t\in \Gamma$  2:$y_{j,t}\leftarrow 0,C_{j}^{ter}\leftarrow 0$, $\forall j\in N_{ter},t\in \Gamma$  3:**for** each t∈Γ **do**  4:   $\Omega_{s}\leftarrow N_{sat}$, $\Omega_{t}\leftarrow N_{ter}$  5:   **while** $\Omega_{s}\neq \emptyset$ and $\sum_{i=1}^{N_{sat}}x_{i,t}<N_{b}$ **do**  6:     **if** $\min_{i\in \Omega_{s}}\left(C_{i}^{sat}/D_{i}^{sat}\right)\leq \min_{j\in \Omega_{t}}\left(C_{j}^{ter}/D_{j}^{ter}\right)$ **then**  7:        $\Theta_{s}\leftarrow \left\{i\in \Omega_{s}|C_{i}^{sat}/D_{i}^{sat}=\min_{k\in \Omega_{s}}\left(C_{k}^{sat}/D_{k}^{sat}\right)\right\}$  8:        $i^{*}\leftarrow$arg$\max_{i\in \Theta_{s}}\left\{D_{i}^{sat}\right\}$  9:        $x_{i^{*},t}\leftarrow 1$10:        $C_{i^{*}}^{sat}\leftarrow C_{i^{*}}^{sat}+{\tilde{c}}_{i^{*}}^{sat}$11:        $\Omega_{s}\leftarrow \left\{k\in \Omega_{s}|d_{i^{*},k}^{sat}\geq d_{th}^{sat}\right\}$12:        $\Omega_{t}\leftarrow \left\{j\in \Omega_{t}|d_{i^{*},j}^{st}\geq d_{pro}^{st}\right\}$13:     **else**14:        $\Theta_{t}\leftarrow \left\{j\in \Omega_{t}|C_{j}^{ter}/D_{j}^{ter}=\min_{k\in \Omega_{t}}\left(C_{k}^{ter}/D_{k}^{ter}\right)\right\}$15:        $j^{*}\leftarrow$arg$\max_{j\in \Theta_{t}}\left\{D_{j}^{ter}\right\}$16:        $y_{j^{*},t}\leftarrow 1$17:        $C_{j^{*}}^{ter}\leftarrow C_{j^{*}}^{ter}+c_{j^{*}}^{ter}$18:        $\Omega_{t}\leftarrow \Omega_{t}-\left\{j^{*}\right\}$19:        $\Omega_{s}\leftarrow \left\{i\in \Omega_{s}|d_{i,j^{*}}^{st}\geq d_{pro}^{st}\right\}$20:     **end if**21:   **end while**22:   **for** each $j\in \Omega_{t}$ **do**23:     $y_{j,t}\leftarrow 1$24:     $C_{j}^{ter}\leftarrow C_{j}^{ter}+c_{j}^{ter}$25:   **end for**26:**end for**

The computational complexity of the greedy algorithm for MMCDR can be analyzed in a similar way as shown in Section 4.2. Before the resource allocation, it also requires $O(N_{sat})$ and $O(N_{ter})$ operations to calculate the transmission capacity of the satellite terminals and the transmission capacity of the terrestrial systems, respectively. During the resource allocation process, the worst-case complexity is $O(\left(N_{ter}+2N_{b}\right)N_{slot})$ since it requires at most $O(N_{ter}+2N_{b})$ operations to update the candidate sets when one of the time slots in Γ is allocated.

## 6. Simulation Results

Consider the downlink transmission scenario in an ISTN, which consists of one LEO satellite system and multiple terrestrial systems. The LEO satellite system consists of an LEO satellite and $N_{sat}=16$ satellite terminals. Within the footprint of the satellite, the locations of the satellite terminals are generated by a Poisson point process [30]. The inter-system protection distance $d_{pro}^{st}$ is set to 52 Km, which is equal to the coverage radius of the satellite beam [10,24]. According to the analysis in [23,24], the inter-beam distance threshold $d_{th}^{sat}$ used in the greedy algorithms is set to twice the diameter of the satellite beam, so the CCI among the satellite beams is negligible. There are $N_{ter}=16$ terrestrial systems within the footprint of the satellite. The locations of the terrestrial BSs are modeled by an MHP [30]. The path loss model and the shadowing standard deviation in the urban microcell (UMi) line-of-sight (LOS) scenario are considered in the terrestrial systems. Assuming $d_{j}^{ter}$ denotes the distance between the terrestrial BS and the terrestrial terminal in the *j*-th terrestrial system, the path loss in dB can be expressed as [32]

(32) $$L_{j}^{ter}\left(\mathrm{dB}\right)=32.4+21\log_{10}\left(d_{j}^{ter}\right)+20\log_{10}\left(f\right).$$

The UMi cell radius is typically set to 500 m [32]. The capacity requirements and the average traffic demands of the satellite terminals and the terrestrial systems are independent random variables that follow the uniform distribution. These random variables are independently generated, which makes the distribution of the capacity requirements and the traffic demands uneven. The simulation parameters are summarized in Table 1.

### 6.1. Simulation Results for MWSC

To evaluate the performance of the proposed resource allocation schemes for MWSC, the following schemes are used as the baselines in the simulations:

- The resource allocation scheme aiming at maximizing the total capacity of the satellite system (MTCS) [23]. The satellite allocates the time slots according to the requested capacity of the satellite terminals, and the terrestrial BSs outside the dynamical protection zones can perform transmission. The terrestrial BSs can determine whether they are located in the dynamical protection zones by receiving the resource allocation results broadcasted by the satellite or by the spectrum sensing results.
- The random resource allocation (RRA) scheme. The satellite randomly selects $N_{b}$ from $N_{sat}$ satellite terminals to provide services in each time slot [21], and the terrestrial BSs outside the dynamical protection zones can perform transmission.
- The dedicated resource allocation (DRA) scheme. Dedicated frequency bands are allocated to the satellite system and the terrestrial systems, respectively. The bandwidth of the dedicated frequency band is equal to half of the full transmission bandwidth. The MTCS scheme is adopted in the satellite system, and the terrestrial BSs can perform transmission continuously.

**Figure 2:**
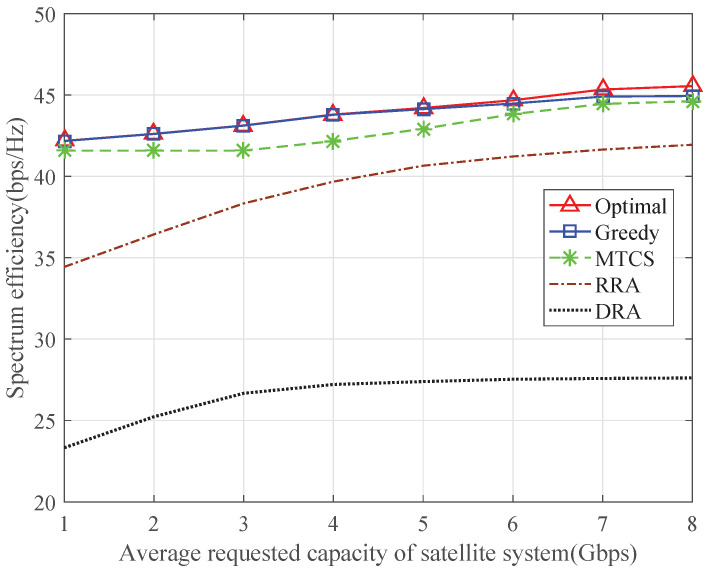
Spectrum efficiency of ISTN with varying requested capacity of satellite system.

Figure 2 depicts the spectrum efficiency of the ISTN as a function of the average total requested capacity of the satellite system in gigabits per second (Gbps). The average total requested capacity of the satellite system is the sum of the average requested capacity of each satellite terminal, and the average minimum required capacity of each terrestrial system is 650 megabits per second (Mbps). The proposed optimal algorithm and greedy algorithm outperform the other schemes since they consider the capacity requirements of both the satellite and terrestrial systems. The performance of the greedy algorithm is almost the same as that of the optimal algorithm when the average requested capacity is relatively low, and it is a bit lower than that of the optimal algorithm with the increase of the requested capacity. The MTCS scheme cannot achieve the optimum spectrum efficiency in ISTN since the capacity requirements of the terrestrial systems are not considered. The RRA scheme is unable to flexibly schedule the resources according to the capacity requirements, so its performance is relatively low. When the satellite and terrestrial systems use the dedicated frequency band with half of the bandwidth, the spectrum efficiency of the ISTN degrades significantly, which verifies the effectiveness of the spectrum sharing scheme in improving the spectrum efficiency.

**Figure 3:**
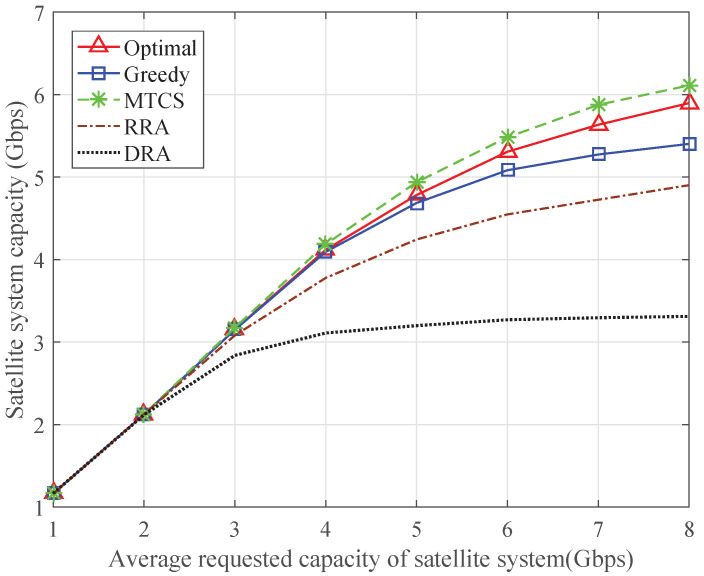
Satellite system capacity with varying requested capacity of satellite system.

**Figure 4:**
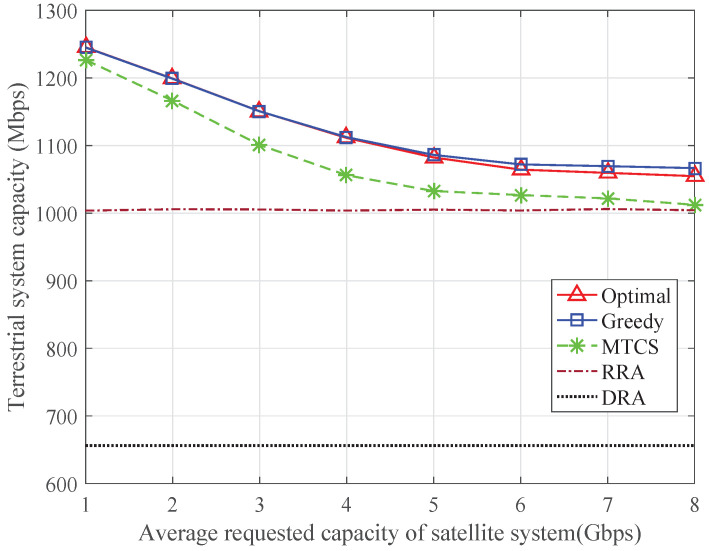
Terrestrial system capacity with varying requested capacity of satellite system.

To further demonstrate the characteristics of different schemes, Figure 3 and Figure 4 show the total transmission capacity of the satellite system and the average transmission capacity of each terrestrial system, respectively. As shown in Figure 3, when the requested capacity of the satellite system is low, the satellite system capacity increases linearly since all the capacity requirements can be met. However, because of the limited transmission resources of the satellite system, the satellite system capacity tends to saturate as the requested capacity increases, and the performance gap among different schemes becomes larger. The satellite system capacity of the MTCS scheme is higher than that of the other schemes, but the transmission capacity of the terrestrial systems cannot be guaranteed. The satellite system capacity of the proposed optimal algorithm is close to that of the MTCS scheme, while the average terrestrial system capacity is obviously improved. Owing to the introduction of the resource guarantee factor *Q*, the greedy algorithm can achieve higher terrestrial system capacity than the optimal algorithm. The RRA scheme takes no account of the capacity requirements, so the average transmission capacity of each terrestrial system remains unchanged, and the resources allocated to each satellite terminal cannot match its capacity requirement. When the DRA scheme is adopted, both the satellite and terrestrial systems cannot achieve the desired capacity because of the lack of available resources.

**Figure 5:**
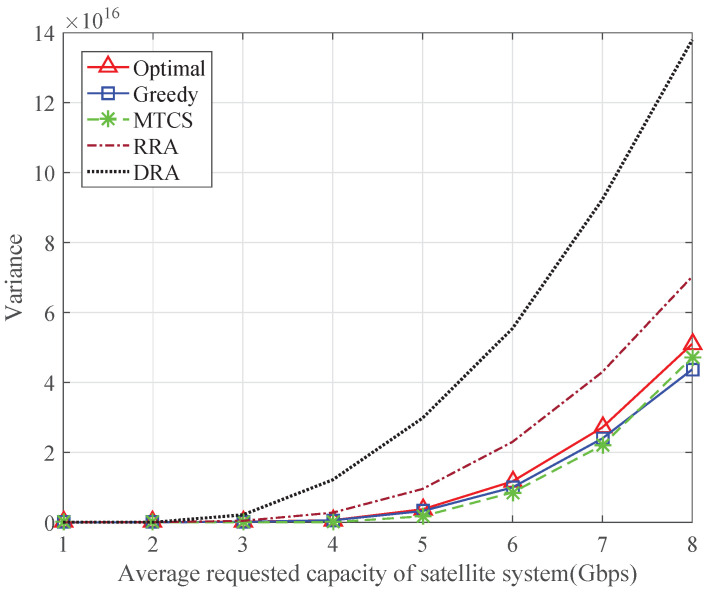
The variance between the requested capacity and the transmission capacity in the satellite system.

Figure 5 shows the sum of the variance between the requested capacity and the transmission capacity of each satellite terminal during one second. The variances of the proposed algorithms are always lower than those of the RRA and DRA schemes. This is because the proposed algorithms can allocate an appropriate number of resources to the satellite terminals, which implies that the algorithms can achieve better matching between the requested capacity and the transmission capacity. The performance of the proposed algorithms is close to that of the MTCS scheme, especially the greedy algorithm. As mentioned in Section 4.2, the greedy algorithm gives priority to the satellite terminals with higher remaining requested capacity during the resource allocation process, so the transmission capacity of each satellite terminal is closer to its requested capacity.

**Figure 6:**
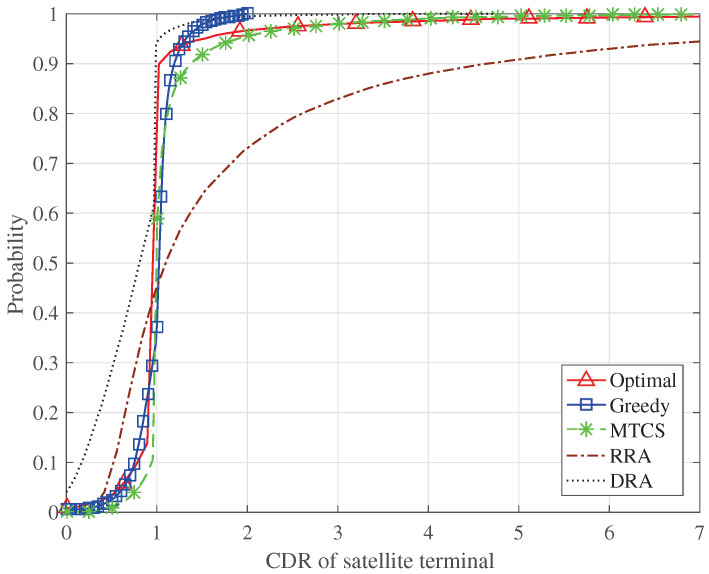
CDF of the CDR of satellite terminal.

Figure 6 presents the cumulative distribution function (CDF) of the CDR of the satellite terminal, i.e., $C_{i}^{sat}/R_{i}^{sat}$. Since the optimization objective of P1 in (11) depends on the minimum value of $C_{i}^{sat}$ and $R_{i}^{sat}$, the transmission capacity allocated to most of the satellite terminals is relatively close to their requested capacity when the proposed algorithms and the MTCS scheme are adopted. As shown in Algorithm 1, the greedy algorithm no longer allocates the time slots to the satellite terminals if their requested capacities have been met, so almost all satellite terminals have the CDRs less than two, which relieves the resource waste. The RRA scheme does not schedule the resources according to the requested capacity of the satellite terminals, which results in a larger number of satellite terminals with high CDRs and a larger number of satellite terminals with low CDRs. Because of the halving of the transmission bandwidth, the DRA scheme can only meet the requested capacity of about 40% of the satellite terminals.

Table 2 presents the satisfaction of the terrestrial systems with varying average total requested capacity of the satellite system. Satisfaction is defined as the proportion of the terrestrial systems whose minimum capacity requirements are satisfied. Because of constraint (15), the proposed optimal algorithm and greedy algorithm can provide enough transmission resources to the terrestrial systems, so the satisfaction is always 100%. As the average requested capacity increases, the MTCS scheme allocates more time slots to the satellite terminals, so the satisfaction of the terrestrial systems gradually decreases. The satisfaction of the RRA scheme is around a certain value since it is not affected by the change in the capacity requirements. When using the dedicated frequency band, the available resources are fixed and limited, so about half of the terrestrial systems cannot achieve their minimum required capacity.

### 6.2. Simulation Results for MMCDR

To evaluate the performance of the proposed resource allocation schemes for MMCDR, the following schemes are used as the baselines in the simulations:

- The resource allocation scheme aiming at maximizing the minimum CDR of the satellite system (MMCS) [25]. The satellite allocates the time slots according to the average traffic demands of the satellite terminals, and the terrestrial BSs outside the dynamical protection zones can perform transmission.
- The RRA scheme.
- The DRA scheme, where the MMCS scheme is adopted in the satellite system with half of the bandwidth.

**Figure 7:**
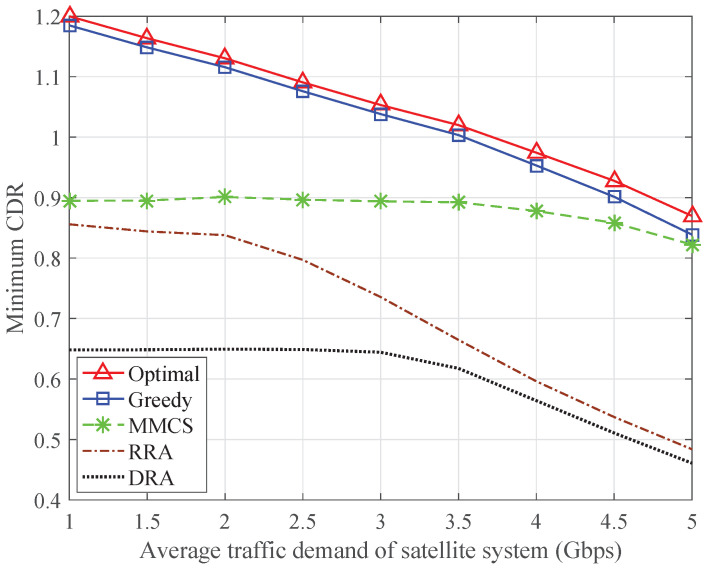
Minimum CDR in the ISTN with varying traffic demand of the satellite system.

Figure 7 depicts the minimum CDR in the ISTN as a function of the average total traffic demand of the satellite system in Gbps. The average total traffic demand of the satellite system is the sum of the average traffic demand of each satellite terminal, and the average traffic demand of each terrestrial system is evenly distributed from 250 to 1050 Mbps. The minimum CDR of the proposed optimal algorithm is always higher than that of the other schemes since it provides the optimal resource allocation results for the ISTN. Regardless of the change in the traffic demand, the gap between the minimum CDRs of the greedy algorithm and the optimal algorithm is relatively small. The MMCS, RRA, and DRA schemes cannot achieve the desirable performance since part of the terrestrial BSs or satellite terminals has not been allocated enough transmission resources.

**Figure 8:**
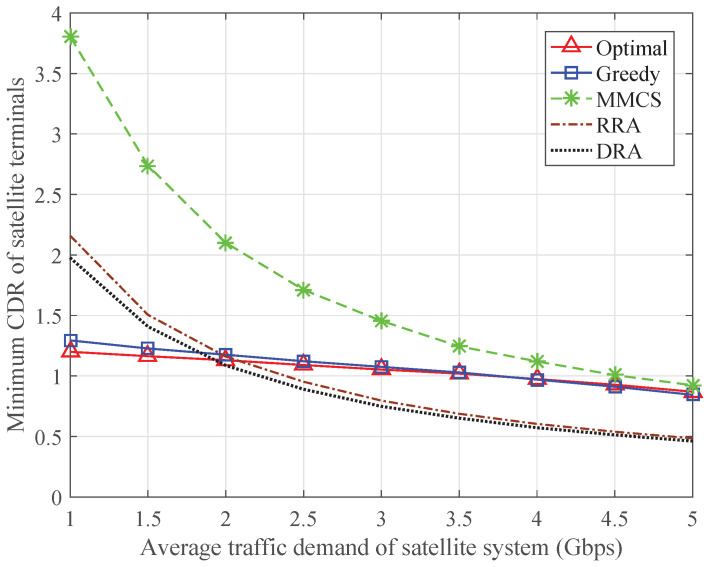
Minimum CDR of the satellite terminals with varying traffic demand of the satellite system.

**Figure 9:**
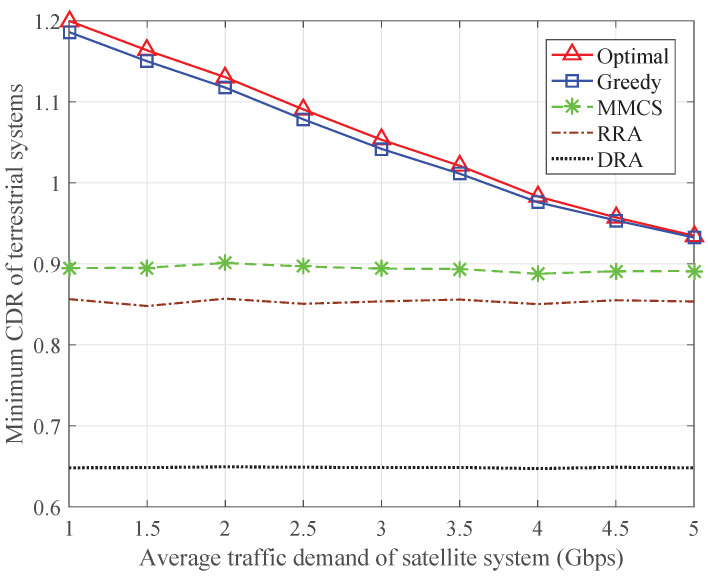
Minimum CDR of the terrestrial systems with varying traffic demand of the satellite system.

To reveal the intrinsic factors affecting the minimum CDR in the ISTN, Figure 8 and Figure 9 show the minimum CDR of the satellite terminals and the minimum CDR of the terrestrial systems, respectively. The MMCS scheme can maximize the minimum CDR of the satellite terminals, but the minimum CDR in the ISTN is mainly limited by the minimum CDR of the terrestrial systems. The RRA scheme and the DRA scheme do not consider the traffic demands of the terrestrial systems, so the minimum CDR of the terrestrial systems remains at a certain value. When the average traffic demand is relatively low, the minimum CDR in the ISTN is subject to the minimum CDR of the terrestrial systems. As the average traffic demand increases, the minimum CDR of the satellite terminals degrades significantly, so the minimum CDR in the ISTN reduces accordingly. By comparison, both the optimal algorithm and the greedy algorithm can keep the minimum CDR of the satellite terminals and the minimum CDR of the terrestrial systems at the same level, which guarantees fairness in the ISTN.

**Figure 10:**
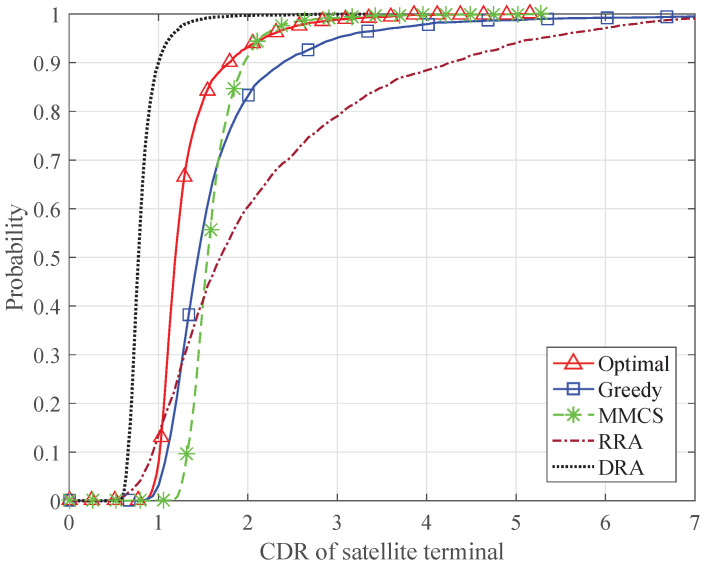
CDF of the CDR of the satellite terminal.

**Figure 11:**
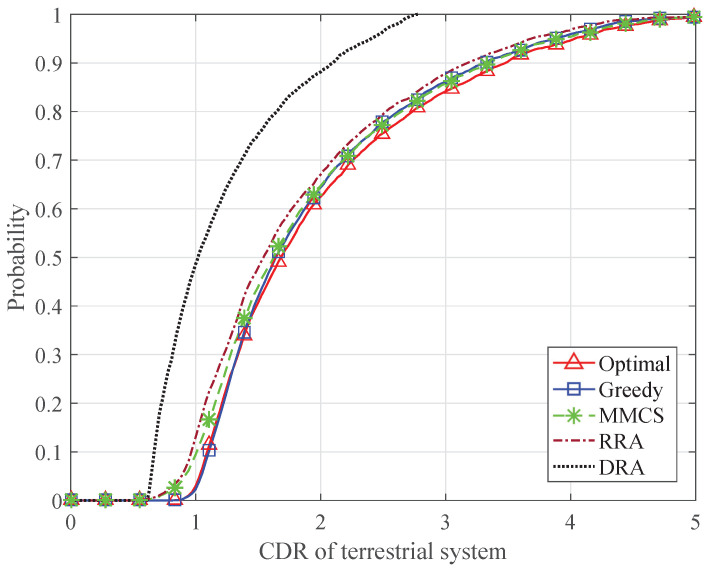
CDF of the CDR of the terrestrial system.

Furthermore, Figure 10 and Figure 11 present the CDF of the CDR of the satellite terminal and the CDR of the terrestrial system, respectively. The average total traffic demand of the satellite system is set to 3 Gbps. Because the MMCS scheme only considers the traffic demands of the satellite terminals, the CDRs of the satellite terminals are controlled within a desirable range, while some of the terrestrial systems cannot obtain enough transmission resources. The RRA scheme results in large differences among the CDRs of the satellite terminals because of the mismatch between the transmission capacity and traffic demands. When the DRA scheme is adopted, the available resources of each system are limited, which leads to the lower CDRs of the satellite terminals and the terrestrial systems. By allocating an appropriate number of time slots to the satellite terminals and the terrestrial BSs, the proposed algorithms can keep all the CDRs within a desirable range, which ensures fairness among all the satellite terminals and the terrestrial systems. Compared with the optimal algorithm, the greedy algorithm results in more satellite terminals with high CDR. This is because the greedy algorithm continuously allocates each time slot to the candidate satellite terminals until the candidate set is empty or $N_{b}$ satellite terminals have been allocated.

### 6.3. Comparison of Computational Complexity

**Figure 12:**
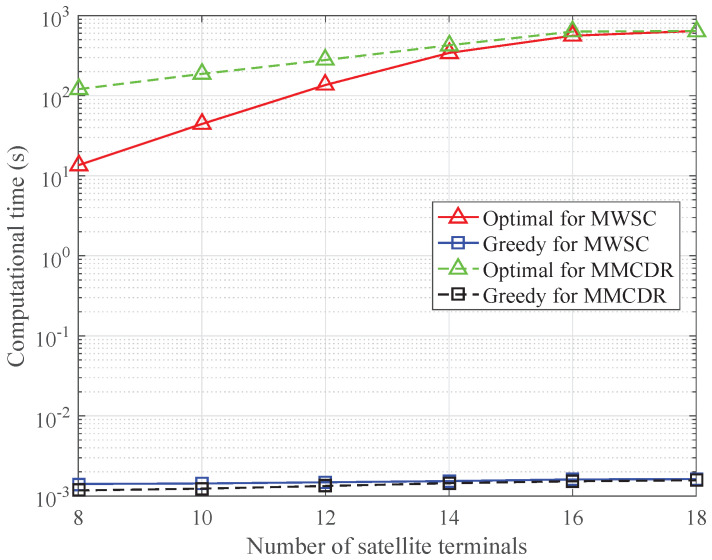
Computational time with varying numbers of satellite terminals.

In order to evaluate the computational complexity of the proposed algorithms, Figure 12 compares the average computational time of the optimal and greedy algorithms for MWSC and MMCDR. The average total requested capacity and the average total traffic demand of the satellite system are set to 5 Gbps, and the minimum required capacity and the average traffic demand of each terrestrial system are evenly distributed from 250 to 1050 Mbps. When solving problems P1’ and P2’ by the optimal algorithms proposed in Section 4.1 and Section 5.1, the computational time increases dramatically with the number of satellite terminals. By comparison, the computational time of the greedy algorithms is insensitive to the number of satellite terminals and is four to five orders of magnitude lower than that of the optimal algorithms. The simulation results in Section 6.1 and Section 6.2 show that the spectrum efficiency of the greedy algorithm for MWSC and the minimum CDR of the greedy algorithm for MMCDR are close to those of the optimal algorithms. Therefore, the proposed greedy algorithms can be adopted to achieve desirable performance with much lower computational complexity, which is attractive for real-time resource scheduling in the ISTN.

## 7. Conclusions

In this paper, we have investigated the BH-based resource allocation scheme for the ISTN in the spectrum sharing scenario. To avoid inter-system CCI and improve the reusability of the frequency resources, a BH-based spectrum sharing scheme for the ISTN has been presented, where dynamical protection zones are constructed according to the beam allocation pattern of the satellite. Based on the spectrum sharing scheme, the resource allocation problems for MWSC and MMCDR have been formulated to improve the spectrum efficiency and guarantee fairness, respectively. Optimal solutions have been obtained by reformulating the problems as MILP problems. In order to solve problems with low computational complexity, two greedy suboptimal algorithms have been proposed for MWSC and MMCDR, respectively. The simulation results show that the proposed resource allocation algorithms achieve higher spectrum efficiency in the ISTN and meet the unevenly distributed traffic demands of both the satellite and terrestrial systems. It has also shown that both the greedy algorithms with low complexity perform closely to the optimal algorithms, which are more suitable for real-time resource scheduling in the ISTN. Our future work is to evaluate the impact of the inaccurate location information and capacity requirements on the performance of the proposed algorithms and make corresponding improvements to the resource allocation scheme for ISTN.

## Figures and Tables

**Table 1:** Simulation parameters.

Parameter	Symbol	Value
ISTN common parameters		
Carrier frequency	*f*	19.5 GHz
Transmission bandwidth	*W*	500 MHz
Number of time slots in a BH time window	$N_{slot}$	50
Time slot period	$T_{slot}$	1 ms
Inter-system protection distance	$d_{pro}^{st}$	52 Km
Weighting factor	γ	0.1
Satellite system parameters		
Satellite orbit height	$h_{s}$	1000 Km
Number of satellite terminals	$N_{sat}$	16
Number of spot beams	$N_{b}$	4
Transmit power per beam	$P_{b}^{sat}$	4 dBw
Boltzmann constant	*K*	$1.38\times 10^{-23}$ W/(Hz·K)
Receiver noise temperature	$T_{n}$	293 K
Peak transmitting antenna gain	$G_{T,0}^{sat}$	31 dBi
3 dB beam width of the transmitting antenna	$\theta_{3dB}$	2.98°
Peak receiving antenna gain	$G_{R,0}^{sat}$	36 dBi
inter-beam distance threshold	$d_{th}^{sat}$	200 Km
Terrestrial system parameters		
Number of terrestrial terminals	$N_{ter}$	16
Transmit power per BS	$P_{j}^{ter}$	0 dBw
Noise power	$N_{0}^{ter}$	−113 dBw
Transmitting antenna gain	$G_{T}^{ter}$	10 dBi
Receiving antenna gain	$G_{R}^{ter}$	3 dBi
Scenario of path loss model		UMi LOS
Shadowing standard deviation		4 dB
UMi cell radius	$d_{j}^{ter}$	500 m
Minimum separation distance	δ	52 Km

**Table 2:** The satisfaction of the terrestrial systems.

Requested Capacity	Optimal	Greedy	MTCS	RRA	DRA
1 Gbps	100%	100%	99.90%	88.54%	49.98%
2 Gbps	100%	100%	98.53%	88.87%	49.89%
3 Gbps	100%	100%	94.69%	88.59%	49.77%
4 Gbps	100%	100%	91.16%	88.60%	49.98%
5 Gbps	100%	100%	89.53%	88.83%	50.02%
6 Gbps	100%	100%	88.43%	88.57%	50.04%
7 Gbps	100%	100%	86.09%	88.82%	49.82%
8 Gbps	100%	100%	85.56%	88.95%	50.10%

## Data Availability

The original contributions presented in the study are included in the article; further inquiries can be directed to the corresponding author.

## Abbreviations

The following abbreviations are used in this manuscript:

6G	Sixth-generation
BH	Beam-hopping
BS	Base station
CCI	Co-channel interference
CDF	Cumulative distribution function
CDR	Capacity to demand ratio
CR	Cognitive radio
DRA	Dedicated resource allocation
EZ	Exclusion zone
Gbps	Gigabits per second
ISTN	Integrated satellite-terrestrial network
LEO	Low Earth orbit
LOS	Line of sight
Mbps	Megabits per second
MEO	Medium earth orbit
MHP	Matern hardcore process
MILP	Mixed-integer linear programming

MMCDR	Maximizing minimum capacity to demand ratio
MMCS	Maximizing minimum CDR of satellite system
MTCS	Maximizing total capacity of satellite system
MWSC	Maximizing weighted sum of capacity
NCC	Network control center
RRA	Random resource allocation
SINR	Signal-to-interference-plus-noise ratio
UMi	Urban microcell

## References

[1] Zhang Z., Xiao Y., Ma Z., Xiao M., Ding Z., Lei X., Karagiannidis G.K., Fan P. (2019). 6G wireless networks: Vision, requirements, architecture, and key technologies. IEEE Veh. Technol. Mag..

[2] Liu J., Shi Y., Fadlullah Z.M., Kato N. (2018). Space-air-ground integrated network: A survey. IEEE Commun. Surveys Tuts..

[3] Ding F., Bao C., Zhou D., Sheng M., Shi Y., Li J. (2024). Toward autonomous resource management architecture for 6G satellite-terrestrial integrated networks. IEEE Netw..

[4] Zhang Y., Zhang H., Zhou H., Long K., Karagiannidis G.K. (2022). Resource allocation in terrestrial-satellite-based next generation multiple access networks with interference cooperation. IEEE J. Sel. Areas Commun..

[5] Zhang M., Yang X., Bu Z. Joint channel selection and spectrum sensing in integrated satellite-terrestrial networks. Proceedings of the 2023 IEEE 24th International Workshop on Signal Processing Advances in Wireless Communications (SPAWC).

[6] Lee H.-W., Medles A., Chen C.-C., Wei H.-Y. (2023). Feasibility and opportunities of terrestrial network and non-terrestrial network spectrum sharing. IEEE Wireless Commun..

[7] Tang W., Thompson P., Evans B. Frequency sharing between satellite and terrestrial systems in the Ka band: A database approach. Proceedings of the 2015 IEEE International Conference on Communications (ICC).

[8] Chen Z., Yang J. A database-assisted spectrum sharing for satellite system and terrestrial cellular network. Proceedings of the 2019 IEEE 5th International Conference on Computer and Communications (ICCC).

[9] Zhang C., Jiang C., Kuang L., Jin J., He Y., Han Z. (2019). Spatial spectrum sharing for satellite and terrestrial communication networks. IEEE Trans. Aerosp. Electron. Syst..

[10] Yang M., Guan X., Miao X. Spectrum sharing schemes in integrated satellite-terrestrial network. Proceedings of the 37th International Communications Satellite Systems Conference (ICSSC-2019).

[11] Liu X., Lam K.-Y., Li F., Zhao J., Wang L., Durrani T.S. (2021). Spectrum sharing for 6G integrated satellite-terrestrial communication networks based on NOMA and CR. IEEE Netw..

[12] Wang J., Guo D., Zhang B., Jia L., Tong X. (2019). Spectrum access and power control for cognitive satellite communications: A game-theoretical learning approach. IEEE Access.

[13] Ruan Y., Li Y., Zhang R., Jiang L. (2022). Energy efficient power control for cognitive multibeam-satellite terrestrial networks with poisson distributed users. IEEE Trans. Cogn. Commun. Netw..

[14] Zuo P., Peng T., Linghu W., Wang W. (2018). Resource allocation for cognitive satellite communications downlink. IEEE Access.

[15] Li T., Yao R., Fan Y., Zuo X., Miridakis N.I., Tsiftsis T.A. (2023). Pattern design and power management for cognitive LEO beaming hopping satellite-terrestrial networks. IEEE Trans. Cogn. Commun. Netw..

[16] Zhang X., Guo D., An K., Zheng G., Chatzinotas S., Zhang B. (2021). Auction-based multichannel cooperative spectrum sharing in hybrid satellite-terrestrial IoT networks. IEEE Internet Things J..

[17] Zhang X., Guo D., An K., Chen Z., Zhao B., Ni Y., Zhang B. (2019). Performance analysis of NOMA-based cooperative spectrum sharing in hybrid satellite-terrestrial networks. IEEE Access.

[18] Liang Y.-C., Tan J., Jia H., Zhang J., Zhao L. (2021). Realizing intelligent spectrum management for integrated satellite and terrestrial networks. J. Commun. Inf. Netw..

[19] Martikainen H., Majamaa M., Puttonen J. Coordinated dynamic spectrum sharing between terrestrial and non-terrestrial networks in 5G and beyond. Proceedings of the 2023 IEEE 24th International Symposium on a World of Wireless, Mobile and Multimedia Networks (WoWMoM).

[20] Zhang M., Yang X., Bu Z. Resource allocation with interference avoidance in beam-hopping based LEO satellite systems. Proceedings of the 2023 4th Information Communication Technologies Conference (ICTC).

[21] Hu X., Zhang Y., Liao X., Liu Z., Wang W., Ghannouchi F.M. (2020). Dynamic beam hopping method based on multi-objective deep reinforcement learning for next generation satellite broadband systems. IEEE Trans. Broadcast..

[22] Wang L., Zhang C., Qu D., Zhang G. Resource allocation for beam-hopping user downlinks in multi-beam satellite system. Proceedings of the 2019 15th International Wireless Communications & Mobile Computing Conference (IWCMC).

[23] Wang Y., Bian D., Hu J., Tang J., Wang C. A flexible resource allocation algorithm in full bandwidth beam hopping satellite systems. Proceedings of the 2019 IEEE 3rd Advanced Information Management, Communicates, Electronic and Automation Control Conference (IMCEC).

[24] Tang J., Bian D., Li G., Hu J., Chen J. (2021). Resource allocation for LEO beam-hopping satellites in a spectrum sharing scenario. IEEE Access.

[25] Lei L., Lagunas E., Yuan Y., Kibria M.G., Chatzinotas S., Ottersten B. (2020). Beam illumination pattern design in satellite networks: Learning and optimization for efficient beam hopping. IEEE Access.

[26] Xiao A., Chen Z., Wu S., Jin S., Ma L. (2022). Collaborative long-short term bandwidth allocation for satellite-terrestrial networks. IEEE Commun. Lett..

[27] Yang H., Yang D., Li Y., Kuang J. (2023). Cluster-based beam hopping for energy efficiency maximization in flexible multibeam satellite systems. IEEE Commun. Lett..

[28] Gao R., Wang K., Lin W., Kang H. Joint beam-hopping pattern scheduling and power allocation for LEO satellite network. Proceedings of the 2024 IEEE Wireless Communications and Networking Conference (WCNC).

[29] Zhang C., Jin J., Zhang H., Li T. (2018). Spectral coexistence between LEO and GEO satellites by optimizing direction normal of phased array antennas. China Commun..

[30] Cho S.-R., Choi W. (2014). Coverage and load balancing in heterogeneous cellular networks with minimum cell separation. IEEE Trans. Mobile Comput..

[31] Caini C., Corazza G.E., Falciasecca G., Ruggieri M., Vatalaro F. (1992). A spectrum-and power-efficient EHF mobile satellite system to be integrated with terrestrial cellular systems. IEEE J. Sel. Areas Commun..

[32] Rappaport T.S., Xing Y., MacCartney G.R., Molisch A.F., Mellios E., Zhang J. (2017). Overview of millimeter wave communications for fifth-generation (5G) wireless networks-with a focus on propagation models. IEEE Trans. Antennas Propag..

[33] Thakoor N., Gao J. (2011). Branch-and-bound for model selection and its computational complexity. IEEE Trans. Knowl. Data Eng..

